# Complement C5a induces the generation of neutrophil extracellular traps by inhibiting mitochondrial STAT3 to promote the development of arterial thrombosis

**DOI:** 10.1186/s12959-022-00384-0

**Published:** 2022-04-29

**Authors:** Yejia Chen, Xiaobo Li, Xinxin Lin, Hongbin Liang, Xuewei Liu, Xinlu Zhang, Qiuxia Zhang, Fengyun Zhou, Chen Yu, Li Lei, Jiancheng Xiu

**Affiliations:** Department of Cardiology, State Key Laboratory of Organ Failure Research, Nanfang Hospital, Southern Medical University, Guangzhou, 510515 Guangdong China

**Keywords:** Arterial thrombosis, Complement C5a, Neutrophil extracellular traps, Mitochondrial ROS, Mitochondrial STAT3

## Abstract

**Background:**

Thrombotic events cannot be completely prevented by antithrombotics, implicating a therapeutic gap due to inflammation, a not yet sufficiently addressed mechanism. Neutrophil extracellular traps (NETs) are an essential interface between inflammation and thrombosis, but exactly how the NETotic process is initiated and maintained during arterial thrombosis remains incompletely understood.

**Methods and results:**

We found that the plasma concentrations of C5a were higher in patients with ST-elevation myocardial infarction (STEMI) than in patients with angina and higher in mice with left common carotid artery (LCCA) thrombosis induced by FeCl_3_ than in control mice. We observed that the thrombus area and weight were decreased and that NET formation in the thrombi was reduced in the group treated with the selective C5aR1 receptor inhibitor PMX53 compared with the NaCl group. In vitro, NETosis was observed when C5a was added to neutrophil cultures, and this effect was reversed by PMX53. In addition, our data showed that C5a increased the production of mitochondrial reactive oxygen species (ROS) and that the promotion of NET formation by C5a was mitochondrial ROS (Mito-ROS) dependent. Furthermore, we found that C5a induced the production of Mito-ROS by inhibiting mitochondrial STAT3 activity.

**Conclusions:**

By inhibiting mitochondrial STAT3 to elicit Mito-ROS generation, C5a triggers the generation of NETs to promote the development of arterial thrombosis. Hence, our study identifies complement C5a as a potential new target for the treatment and prevention of thrombosis.

**Supplementary Information:**

The online version contains supplementary material available at 10.1186/s12959-022-00384-0.

## Introduction

Arterial thrombosis is the cause of myocardial infarction (MI) and stroke, which account for the major burden of mortality and disability from cardiovascular events globally [1]. Antithrombosis therapy is the main prevention and treatment but has an inherent risk of bleeding complications [2]. Inflammation and thrombosis are tightly connected processes, contributing to the containment of pathogens via a host defense effector mechanism termed “immunothrombosis” [3]. Due to dysregulation of immunothrombosis, there is likely a key event in the development of thrombotic disorders, including MI and stroke [3]. Thrombotic events cannot be completely prevented by antithrombotics, implicating a therapeutic gap due to inflammation, a not yet sufficiently addressed mechanism [4].

Inflammation was originally characterized by an immune response to pathogenic organisms, where neutrophils are the first cells of the immune system to migrate to and form extracellular chromatin nets decorated with histones and numerous granular proteins to trap bacterial pathogens, namely, neutrophil extracellular traps (NETs) [5, 6]. Considerable recent evidence implicates NETs as a major player in thrombosis, for example, furnishing a scaffold that entraps platelets and erythrocytes [7, 8], expressing or activating coagulation factors (tissue factor, factor XII) [9, 10], and stabilizing fibrin nets [11]. NETs are an essential interface between inflammation and thrombosis. There are some reports on targeting NETs for antithrombotic therapy, showing a reduced thrombus burden [11]. DNase, an enzyme that attacks DNA which serves as the backbone of NETs, and chloroamidine, an inhibitor of peptidylarginine deiminase 4 (PAD4) that has been shown to be critical for NET release, are the main focus of targeting NETs [12, 13]. However, histones and neutrophil elastase (NE) remain after digestion by DNase in NETs and still can promote thrombosis [12]. PAD4 inhibition may attenuate the antibacterial innate immunity mediated by NETs [13], and PAD4 does not play an integral role in nicotinamide adenine dinucleotide phosphate (NADPH) oxidase-dependent NET formation [14, 15]. Various triggers, such as cytokines, bacterial components, the experimental agonist phorbol-12-myristate-13-acetate (PMA), or activated platelets, can stimulate the activation of neutrophils, which leads to the concomitant release of NETs into extracellular compartments [16]. Initiators that trigger NETosis originally in thrombosis could be new options for targeting NETs. However, exactly how the NETotic process is initiated and maintained during arterial thrombosis remains incompletely understood.

Several studies suggest that complement C5a is closely related to thrombosis. In vitro, neutrophil chemotaxis is enhanced by complement C5a in coronary thrombus-derived plasma [17]. In vivo, neutrophils accumulate at the site of thrombus formation and are correlated with C5a and enzymatic infarct size [17]. In a deep vein thrombosis (DVT) model, both thrombotic stability and load are reduced in C5-deficient mice [18]. C5a-mediated neutrophil NET formation has been found in spesis [19], antineutrophil cytoplasmic antibody-associated vasculitis [20], COVID-19 immunothrombosis [21], tumor cell invasion and metastasis [22]. Whether C5a is the major molecule that activates neutrophils to produce NETs, thus promoting the occurrence and development of thrombosis, has not yet been demonstrated. This study was initiated to investigate the relationship between C5a and NETs in arterial thrombosis.

## Materials and methods

### Animals

Eight- to 12-week-old healthy C57BL/6 J mice were purchased from the Laboratory Animal Center of Southern Medical University (Guangzhou, China). All animal protocols were approved by the Animal Research Committee of Southern Medical University and were performed in accordance with the National Institutes of Health Guide for the Care and Use of Laboratory Animals.

### Blood samples collection

Coronary blood samples of fifteen patients with STEMI were collected from Xiangdong Hospital affiliated to Hunan Normal University (10 males, 5 females, Supplemental Table 1). Fifteen angina patients served as the control group (11 males, 4 females, Supplemental Table 2). All samples were collected consecutively in the order of admission. Blood samples were immediately processed after collection. Plasma was obtained by centrifugation at 2,000 rpm for 15 min and stored at -80 °C. This study was approved by the Ethics Committee of Nanfang Hospital of Southern Medical University and the Ethics Committee of the Xiangdong Hospital affiliated to Hunan Normal University. All patients were informed about the experimental nature of this study and gave their consent to participation.

### Ferric chloride (FeCl_3_)-induced thrombosis model

Male C57BL/6 J mice aged 8–12 weeks were used for the FeCl_3_-induced LCCA thrombosis model. The mice were anesthetized with 0.5% pentobarbital sodium via intraperitoneal injection, and a midline incision was made from the manubrium to the hyoid bone. The fascia was bluntly dissected and a section of LCCA at least 5 mm length was isolated. A small plastic piece was placed under the isolated part of the common carotid artery. To induce thrombosis, a piece of filter paper (1 × 2 mm) saturated with 10% (w/v) FeCl_3_ (Sigma, USA) solution was applied to the adventitia for 3 min. The intraperitoneal injection of the selective c5aR1 receptor inhibitor PMX53(1 µg/g [61, 62], Millipore, USA) was performed 30 min before FeCl_3_ exposure. For “rescue” experiments, AG490 (a STAT3 pathway inhibitor, 3 µg/g, MedChemExpress, USA) was injected intraperitoneally 15 min before the injection of PMX53. Thrombi were harvested up to 4–6 h after FeCl_3_ exposure.

#### ELISA

Blood samples collected into EDTA K3 tubes were processed for routine blood counts and to obtain platelet-poor plasma. Plasma concentrations of C5a were determined by a commercially available C5a ELISA kit (Camilo, China). The test was performed as recommended by the manufacturer’s instructions in duplicate.

### Blood flow velocity by Doppler ultrasonography

Doppler ultrasonography was performed on anesthetized mice 4–6 h after FeCl_3_ exposure using a VEVO2100 Imaging System (Visual Sonics, ON, Canada). Firstly, LCCA was located in B-Mode, with the presence of the bifurcation, left internal carotid artery (LICA) and left external carotid artery (LECA) as the standard view. After switching to color Doppler Mode, the region of the LICA 5 mm from the bifurcation was selected as the measurement point, then the mode was switched to pulsed-wave Doppler mode to assess blood flow velocity. Images were acquired for subsequent measurements and calculations.

### Histology and immunofluorescence staining of thrombi

The artery wall containing thrombi were removed and embedded vertically in optimal cutting temperature compound (Sakura, USA). Serial 10-µm frozen sections were cut and placed on slides. For histology, the slides were stained with haematoxylin and eosin (H&E). From each vessel, sections were taken at every 200 μm intervals parallel to the direction of flow. Images were acquired at room temperature with an Olympus BX53 microscope (Center Valley, PA, USA). The area of the thrombus was analysed using ImageJ software (National Institutes of Health, Bethesda, MD). For immunofluorescence staining, samples were incubated with 10% goat serum (Solarbio, China) for 1 h at room temperature. The samples were then incubated overnight at 4 ℃ with rabbit anti-mouse citrullinated histone H3 (CitH3) (1:100, Abcam, ab5103, UK) and rat anti-mouse Ly6G (1:100, BD Pharmingen, Pure 1A8, USA). After washing, the samples were incubated with an Alexa Fluor 594-conjugated goat anti-rabbit IgG antibody (1:200, Bioss, China) and an Alexa Fluor 488-conjugated goat anti-rat IgG antibody (1:200, Biolegend, China) for 1 h at room temperature. The nuclei were labelled with DAPI (BestBio, China). Images were obtained with a Leica (TCS Sp8) confocal microscope (Leica, Germany) at room temperature.

### Neutrophil isolation and NET formation in vitro

Mouse neutrophils were isolated from the bone marrow of tibias and femurs from healthy C57BL/6 J mice using the Neutrophil Isolation Kit (Solarbio, China) following the manufacturer’s instructions. Neutrophils (5 × 10^5^) were incubated with PMX53 (10 μM) or Colivelin (5 μM, Selleck, USA) for 1 h and then stimulated with recombinant murine C5a (0.1 μM, Peprotech, USA) for 3 h. In the AG490-induced NET experiments, neutrophils (5 × 10^5^) were incubated with AG490 (5 μM) for 3 h. The samples were incubated with 10% goat serum for 1 h at room temperature, and then incubated with rabbit anti-mouse CitH3 (1:100, Abcam, ab5103, UK) and rat anti-mouse Ly6G (1:50, BD Biosciences, BV421, clone 1A8, USA) overnight at 4 ℃. After washing, the samples were incubated with an Alexa Fluor 594-conjugated goat anti-rabbit IgG antibody (1:200, Bioss, China) and SYTOX Green Nucleic Acid Stain (167 nM, Thermo, USA). Images were obtained with a Leica (TCS Sp8) confocal microscope (Leica, Germany). For quantification of NET formation in thrombi, 3 distinct criteria needed to be fulfilled: (1) extracellular DNA projections had to emerge, (2) the projections had to originate from cells staining positive for Ly6G, and (3) the structures had to be decorated with CitH3. Only if all of these parameters were met could a structure be defined as NET and included in the quantification.

### Quantifications of NETsin vitro

SYTOX green images of neutrophils were analysed using ImageJ software. The percentage of NET-releasing cells was determined by examining 100–200 cells per sample randomly. Briefly, the DNA area of each cell (circles) was automatically measured in 200-μm^2^ steps, and the distribution of the number of cells across the range of DNA area was obtained. An area larger than 400 μm^2^ was defined as NET. The data were transformed to the percentage of SYTOX-positive cells by dividing the SYTOX-positive counts by the total number of cells as determined from corresponding phase contrast images. The results were plotted as the percentage of cells that were positive for SYTOX for each DNA area range.

### Detection of mitochondria-derived ROS

Mitochondria-derived ROS were detected using MitoSOX Red (Yeasen, Shanghai, China) following the manufacturer’s instructions. Briefly, neutrophils (5 × 10^5^) were incubated in tissue culture plates with MitoTEMPO (10 µM, APExBio, USA) or Colivelin (10 nM) for 1 h. Then, recombinant murine C5a (0.1 μM) was added to the plates, and the cells were incubated for 2 h. After washing, the neutrophils were incubated with probes at 37 ℃ for 10 min and then washed with preheated PBS 3 times for 5 min each time. The Mito-ROS images were obtained with a Leica (TCS Sp8) confocal microscope (Leica, Germany) at room temperature and quantification of the fluorescence signal was performed using ImageJ software.

### Extracellular DNA release measurement by the plate reader assays

For the plate reader assay, neutrophils (3 × 10^4^) were seeded onto 96-well plates in the presence of cell-impermeable SYTOX Green Nucleic Acid Stain (500 nM, Thermo, USA). Fluorescence was measured using a SpectraMax i3X fluorescence microplate reader (Molecular Devices, USA) up to 4 h after the activation of the cells. The fluorescence readout obtained from cells lysed with 0.5% (vol/vol) Triton X-100 (Sigma USA) was considered to represent 100% DNA release, and the NETotic index was calculated as the percentage of the total value.

### Mitochondrial separation

The human HL-60 cell line was purchased from Procell (China), and the cells were incubated with 1.25% dimethyl sulfoxide (DMSO) for 4 days to induce their differentiation into neutrophil-like cells. Recombinant human C5a (0.1 μM, Novoprotein, China) was used to stimulate the neutrophil-like cells for 2 h. Mitochondrial separation was performed using the Mitochondria Isolation Kit (Beyotime, China) according to the manufacturer’s instructions. Briefly, neutrophil-like cells (5 × 10^7^) were centrifuged at 100 g for 10 min at room temperature to collect the cells. Then the cells were homogenized approximately 10–30 times with a homogenizer and centrifuged at 600 g for 10 min at 4 ℃. The obtained supernatant was centrifuged at 11,000 g at 4 ℃ for 10 min, and the pellet was collected as the mitochondrial fraction. Mitochondrial lysis buffer (Beyotime, C3601-4, China) was added to the the pellet, and mitochondrial protein was used for subsequent experiments. Protein concentration of mitochondrial lysates was determined using the Bicinchoninic Acid protein assay (FDbio, Hangzhou, China). The purity of the preparation was verified by immunoblotting.

### Western blotting

An equal amount of mitochondrial protein was subjected to 12% SDS–PAGE and then transferred onto a PVDF membrane (Millipore, Billerica, USA). The membranes were incubated at room temperature for 1 h in blocking buffer. After blocking, the PVDF membranes, which contained the proteins transferred from the gels, were cut into strips (~ 5 mm wide) according to the location (molecular weight) of the protein of interest. Each tailored PVDF membrane strip containing the target protein was then separately incubated over night at 4 ℃ with the corresponding primary antibody: STAT3 rabbit mAb (1:1000, Cell Signaling Technology, Boston, USA), phospho-STAT3 (Ser^727^) rabbit antibody (1:1000, Cell Signaling Technology, Boston, USA), VDAC rabbit mAb (1:1000, Cell Signaling Technology, Boston, USA) and actin mouse mAb (Bioss, Beijing, China). After washing, they were incubated with goat anti-rabbit IgG-HRP (1:5,000, FDbio, Hangzhou, China) or goat anti-mouse IgG-HRP (1:5,000, FDbio, Hangzhou, China). The immunoreactive bands were detected by ECL Substrate (FDbio, Hangzhou, China) and visualized with a GeneGnome XRQ chemiluminescence imager (Syngene, MD, UK). The grey values of the bands were obtained using ImageJ software.

### Statistics

GraphPad Prism (v.8) was used for graphing and statistical analysis. Data are presented as means ± SD (standard deviation). All data performed the test of normal distribution and homogeneity of variance. Two-tailed Student’s t-tests were used for comparisons between two groups. For all experiments involving three or more groups, one-way ANOVA was used. Differences were considered significant at *P* < 0.05.

## Result

### The C5a level was elevated, and NETs participated in arterial thrombosis

**Fig. 1:**
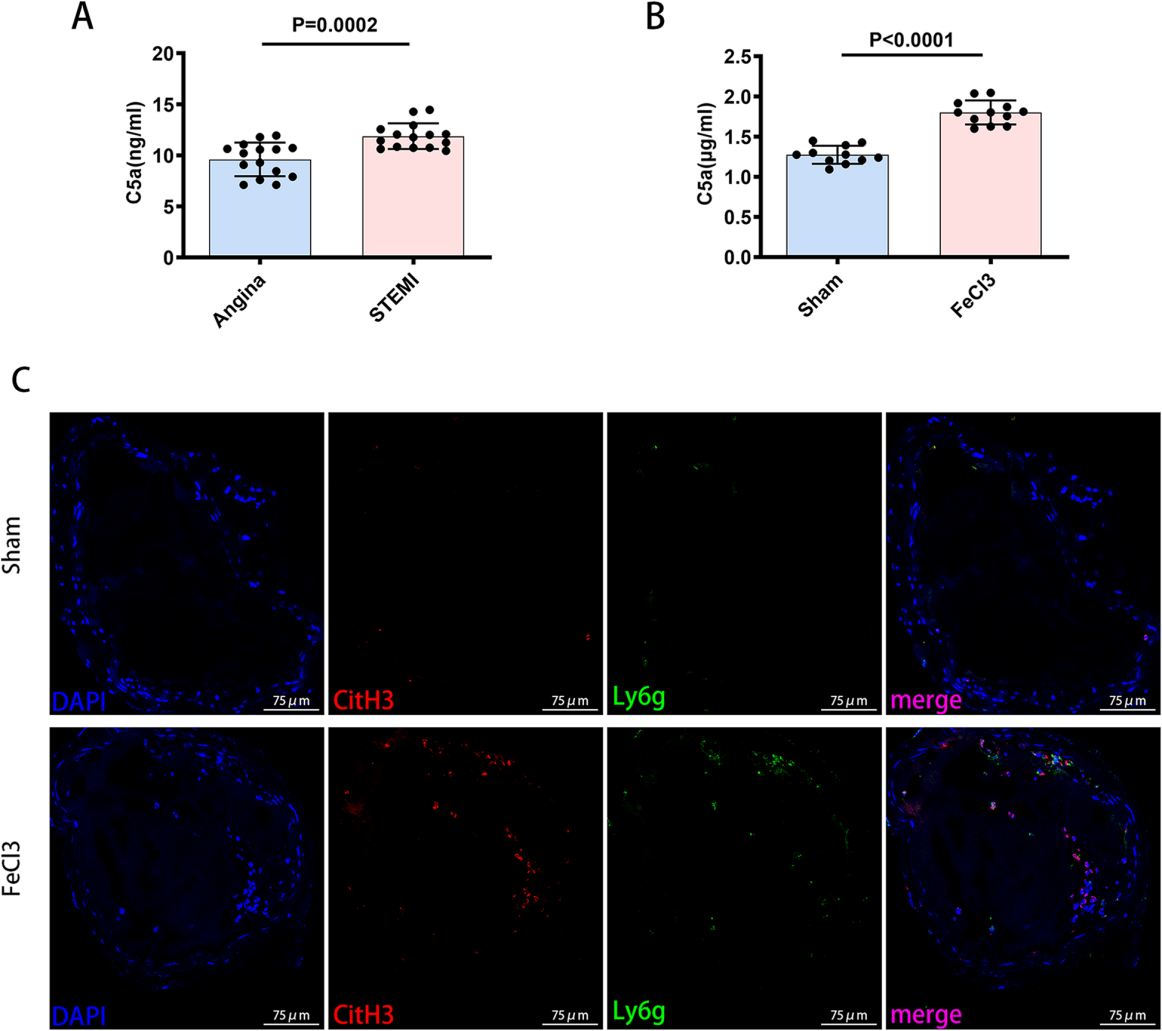
The expression of C5a and NETs in arterial thrombi. **A** ELISA. The concentrations of C5a in plasmas from patients with angina or STEMI (*n* = 15 each). **B** ELISA. The concentrations of C5a in plasmas from C57 BL/6 mice with FeCl_3_-induced arterial thrombosis (*n* = 12) compared with sham mice (*n* = 11). **C** Immunofluorescence staining of cross-sections of the LCCA for CitH3 (red), Ly6g (green) and DAPI (blue) 6 h after FeCl_3_ exposure. Scale bar = 75 µm. Data are presented as mean ± SD

Complement C5a levels have been shown to be elevated at the site of thrombus formation [17]. We compared the plasmas from patients with angina or STEMI, focusing on the level of C5a. Our data confirmed that the plasma concentrations of C5a were higher in patients with STEMI than in patients with angina (Fig. 1A). In addition, the plasma concentrations of C5a were higher in mice with LCCA thrombosis induced by FeCl_3_ than in control mice (Fig. 1B). Accumulating evidence has proved that NETs participate in atherothrombosis [11]. Immunofluorescence showed that NETs participated in the thrombi induced by FeCl_3_ (Fig. 1C).

### C5a triggered neutrophils to release NETs, promoting arterial thrombosis

**Fig. 2:**
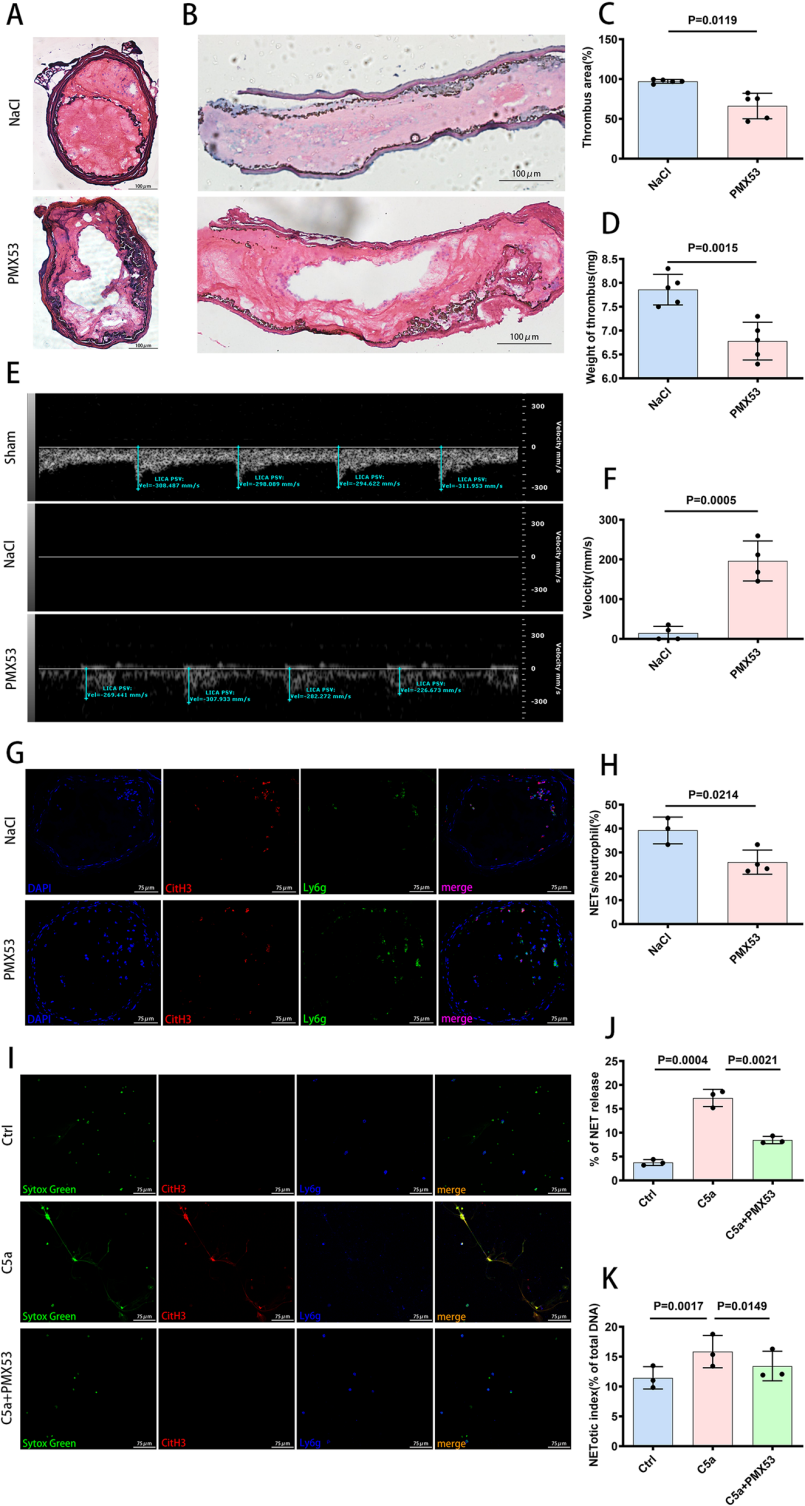
C5a promoted arterial thrombosis by triggering neutrophils to release NETs. **A**, **B** H&E staining of thrombus, cross-sections (**A**) and longitudinal sections (**B**). Scale bar = 100 µm. **C** Quantification of the thrombus size in cross-sections by H&E staining (*n* = 5 each). **D** Thrombus weight (*n* = 5 each). **E** The blood flow velocity in the LICA was dramatically decreased in the FeCl_3_-induced thrombus mice compared with the sham mice, and this change was reversed by PMX53. **F** Quantification of the blood flow velocity (*n* = 4 each). **G** Immunofluorescence staining of cross-sections of the LCCA for CitH3 (red), Ly6g (green) and DAPI (blue) 6 h after FeCl_3_ exposure. Thirty minutes before FeCl_3_ exposure, PMX53 was administered by intraperitoneal injection. Scale bar = 75 µm. **H** Quantification of NET formation capacity shown as the ratio of NETs/neutrophil in LCCA cross-sections subjected to immunofluorescence staining (NaCl group = 3, PMX53 group = 4). **I** Representative images of immunofluorescence stainings of NET formation for DNA (SYTOX green), CitH3 (red), Ly6g(blue) in vitro after stimulation of C5a, and NET formation was attenuated by PMX53. Scale bar = 75 µm. **J** Quantification of NET formation capacity in vitro shown as the percentage of NET release, which was assessed by immunofluorescence staining (*n* = 3 each). (K) NETosis was measured using a plate reader assay. NET release is expressed as percentage of total DNA (*n* = 3 each). Data are presented as mean ± SD

To explore the relationship between C5a and NETs in arterial thrombosis, the selective C5aR1 receptor inhibitor PMX53 was used in the FeCl_3_-induced LCCA thrombosis model. The areas of thrombus in the PMX35 group were decreased compared with those in the NaCl group, which served as a control (Fig. 2A, B, C). Thrombus weight was reduced in C57BL/6 mice injected with PMX53 compared with that in the NaCl control mice (Fig. 2D). As the thrombi induced by FeCl_3_ leaded to occlusion, it is observed that blood flow in the vessel lumen declined or even ceased, and this change was attenuated by PMX53 (Fig. 2E, F). Inhibition of C5aR1 receptor by PMX53 significantly reduced NET formation in thrombi (Fig. 2G, H). In vitro, NETosis was observed when C5a was added to neutrophil cultures, and this effect was reversed by PMX53 (Fig. 2I, J, K). Considering all of the above information, complement C5a promoted arterial thrombosis by stimulating neutrophils to release NETs.

### C5a-promoted release of NETs was dependent on Mito-ROS production

**Fig. 3:**
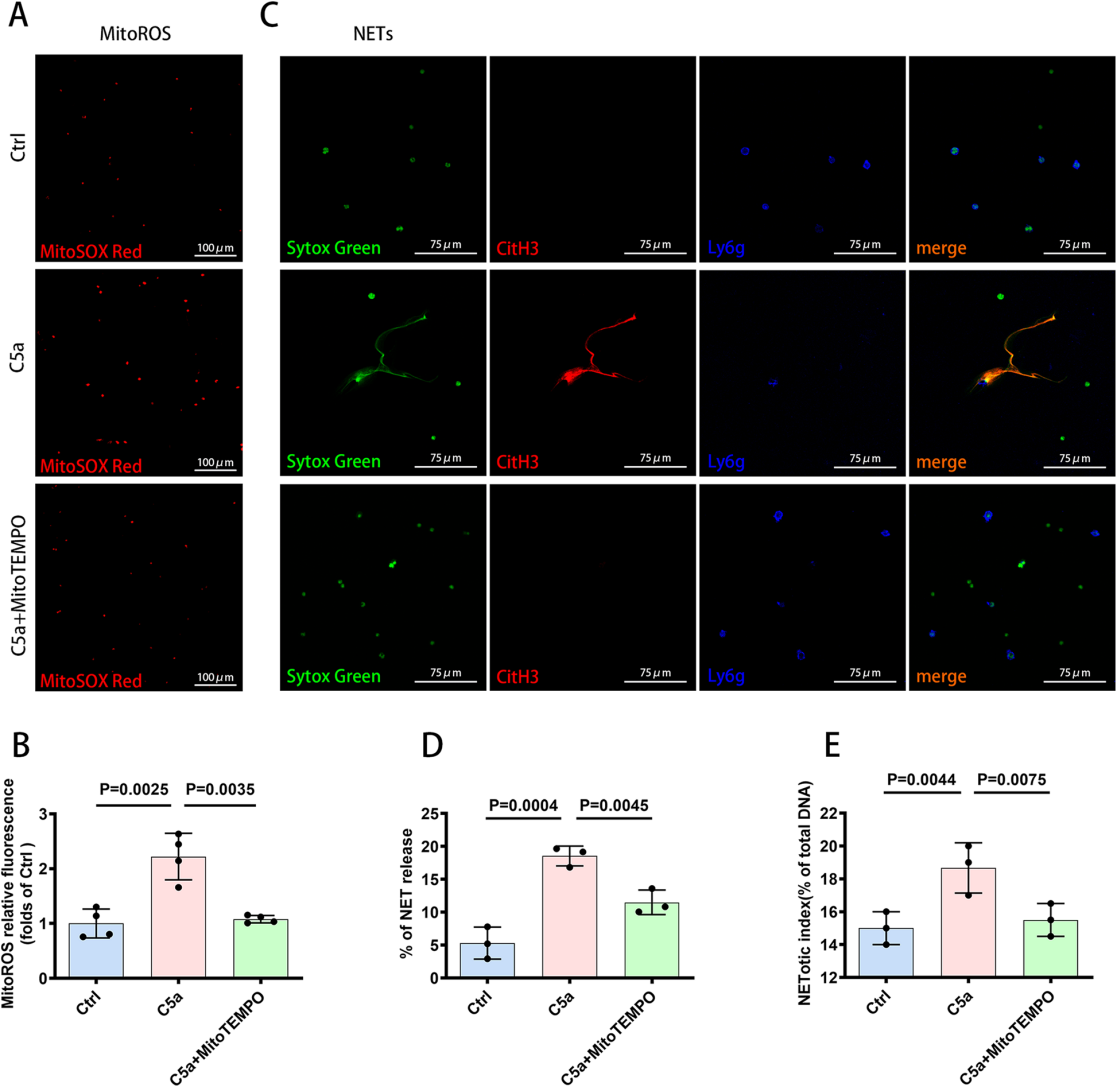
Promotion of NETosis by C5a was Mito-ROS-dependent. **A** Fluorescence images showing ROS production in mitochondria indicated by MitoSox Red dye. Scale bar = 100 µm. **B** Quantification of the production of Mito-ROS shown as MitoROS relative fluorescence (folds of the control group) (*n* = 4 each). **C** Representative images of immunofluorescence staining for DNA (SYTOX green), CitH3 (red), Ly6g (blue) in vitro showing the presence of NETs. Scale bar = 75 µm. **D** Quantification of NET formation capacity shown as percentage of NET release in vitro, as assessed by immunofluorescence staining (*n* = 3 each). **E** NETosis was measured using a plate reader assay, (*n* = 3 each). Data are presented as mean ± SD

The mechanistic details of NETosis are gradually being elucidated, including the processes of NET formation, which is NADPH oxidase-dependent or NADPH oxidase-independent [23, 24]. Different agonists induce either NADPH oxidase-dependent (agonists: PMA, LPS, bacteria, etc.) or NADPH oxidase-independent NET formation (agonists: A23128, ionomycin, uric acid crystals, etc.). These agonists induce different forms of ROS (NADPH oxidase-ROS or Mito-ROS), which are key mediators in the formation of NETs [25]. Whether C5a-promoted release of NETs relies on ROS is unexplored. Our data showed that C5a increased the production of Mito-ROS and that the formation of NETs promoted by C5a was Mito-ROS dependent. Inhibition of Mito-ROS generation with MitoTEMPO reversed the ability of C5a to facilitate the release of NETs (Fig. 3A, B, C, D, E).

### Interactions among C5a, mitochondrial STAT3 and NETs

Signal Transducers and Activators of Transcription family 3 (STAT3) broadly participates in normal development, the acute phase response, chronic inflammation, autoimmunity, metabolism and cancer progression [26]. In addition to being a transcription factor, a small pool of STAT3 is localized in mitochondria, which can reduce the generation of Mito-ROS by positively regulating the mitochondrial electron transport chain (ETC) [27–29]. C5a regulates oxidative stress and causes inflammatory changes through the STAT3 pathway in type 2 diabetic kidney disease [30]. Whether C5a induces the generation of Mito-ROS via mitochondrial STAT3 (Mito-STAT3) has not been detected.

**Fig. 4:**
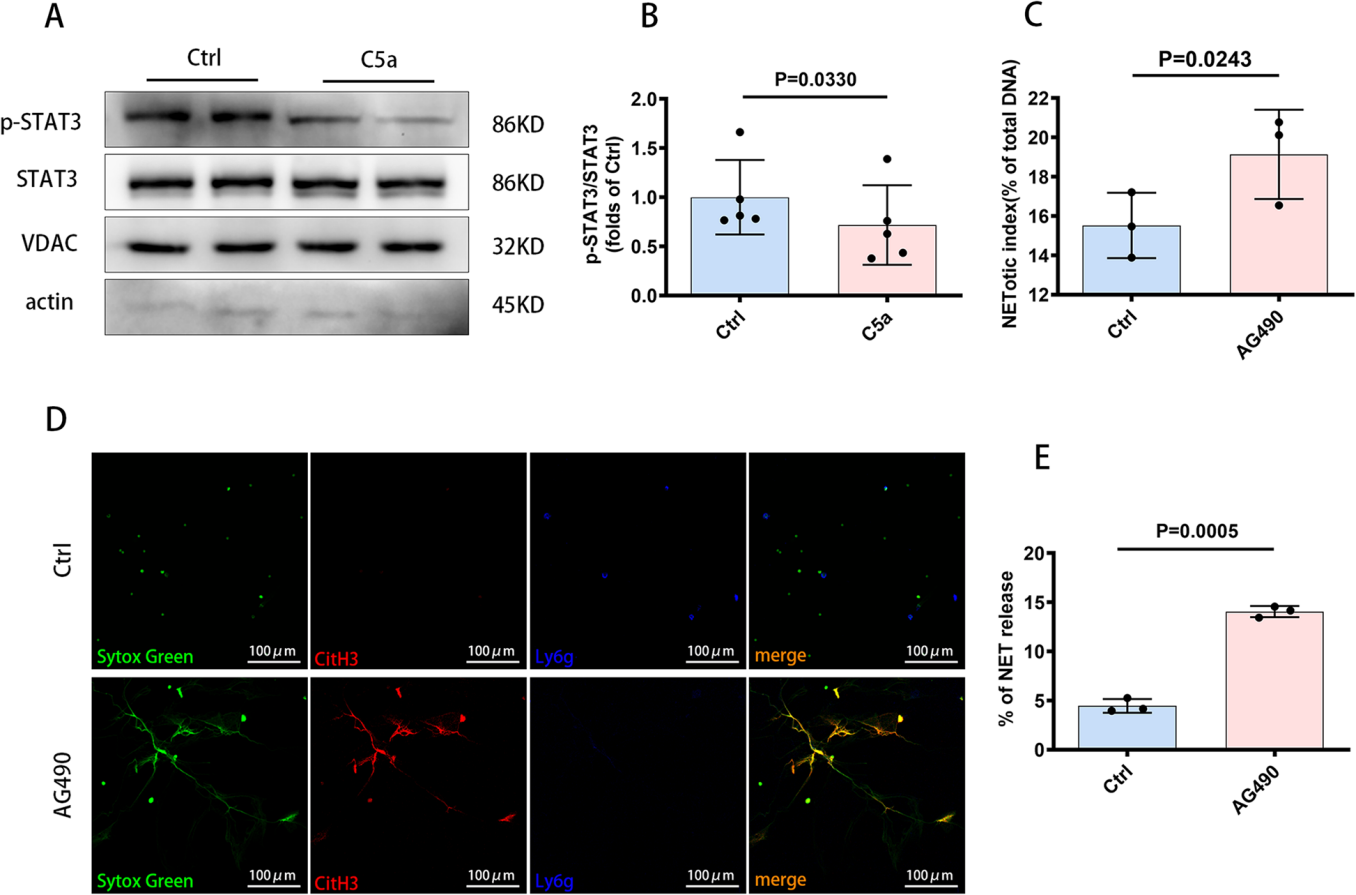
Interactions among C5a, mitochondrial STAT3 and NETs. **A**, **B** Western blot analysis was performed to test the expression levels of mitochondrial STAT3 and p-STAT3 (Ser^727^) in neutrophil-like cells cocultured with C5a, compared with the control. VDAC, a marker of mitochondria, was used as a loading control for mitochondria (*n* = 5 each). **C** NET release in response to buffer or AG490 was measured using a plate reader assay (*n* = 3 each). **D** Representative images of immunofluorescence staining for DNA (SYTOX green), CitH3 (red) and Ly6g (blue) in vitro after stimulation with AG490 showing the presence of NETs. Scale bar = 100 µm. **E** Quantification of NET formation capacity shown as the percentage of NET release in vitro after stimulation with buffer or AG490, as assessed by immunofluorescence staining. (*n* = 3 each). Data are presented as mean ± SD

Human HL-60 cells were incubated with 1.25% DMSO for 4 days to differentiate them into neutrophil-like cells. C5a was used to stimulate neutrophil-like cells, the mitochondria of which were collected for western blot analysis. Compared with that in the control group, the ratio of mitochondrial phospho-STAT3 (Ser^727^) to total mitochondrial STAT3 was lower after stimulation with C5a (Fig. 4A, B). An inhibitor of the STAT3 pathway, AG490, promoted the production of NETs (Fig. 4C, D, E). These results implied that C5a probably induced the NETotic process by inhibiting mitochondrial STAT3 activity.

### *A*G490 abolished the reduction in arterial thrombotic burden induced by PMX53in vivo

**Fig. 5:**
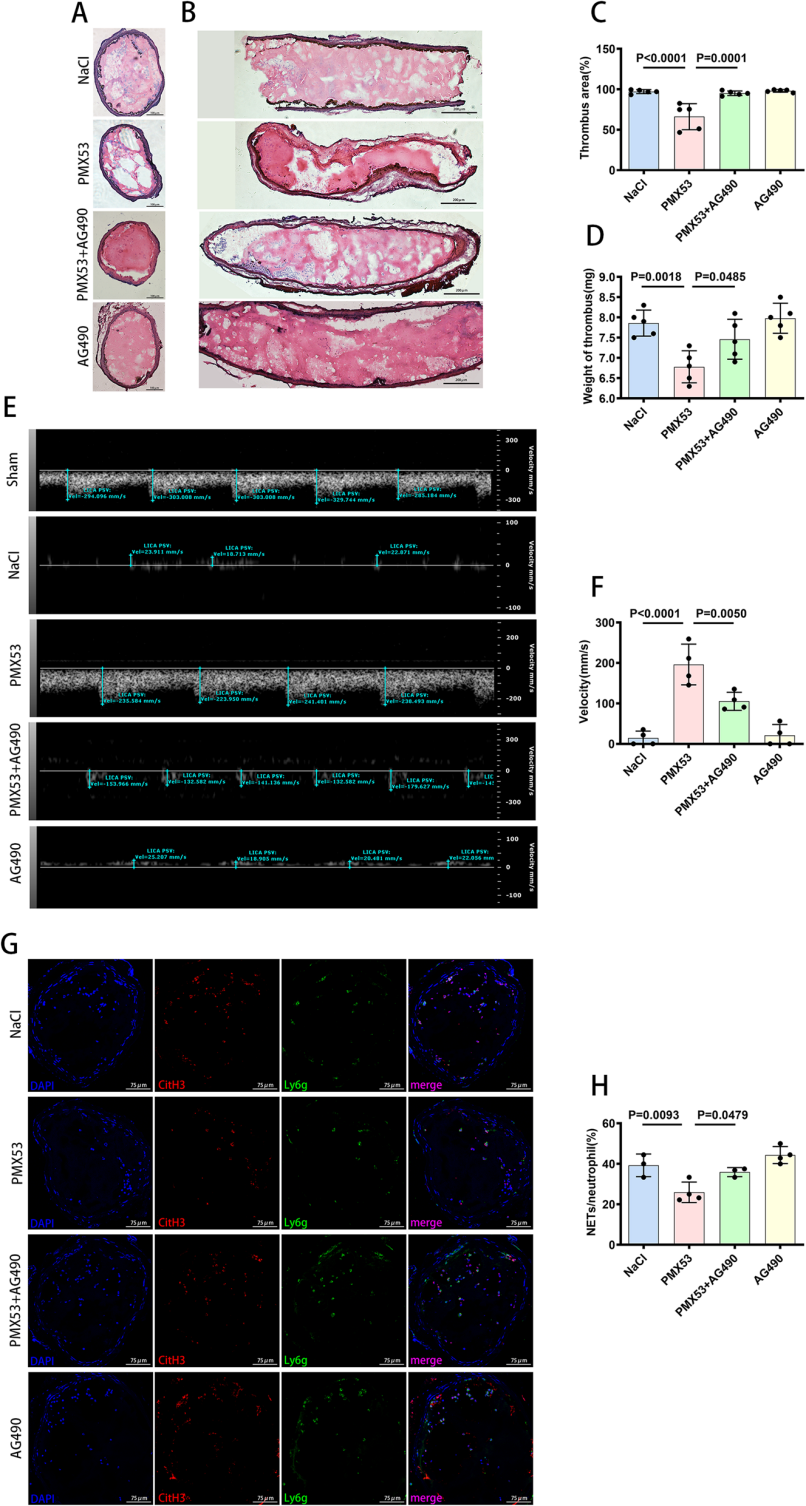
The reduction in arterial thrombotic burden induced by PMX53 was abolished by AG490 in vivo. **A**, **B** H&E staining of thrombus, cross-sections **A** and longitudinal sections **B**. The thrombus area was reduced by PMX53, and AG490 abolished this effect. **A** Scale bar = 100 µm; **B** Scale bar = 200 µm. **C** Quantification of the thrombus size in cross-sections by H&E staining (*n* = 5 each). **D** Thrombus weight (*n* = 5 each). **E** Blood flow velocity in the LICA. AG490 reversed the increased in blood flow induced by PMX53. **F** Quantification of the blood flow velocity (*n* = 4 each). **G** Immunofluorescence staining of cross-sections of the LCCA for CitH3 (red), Ly6g (green) and DAPI (blue) 6 h after FeCl_3_ exposure. Scale bar = 75 µm. **H** Quantification of NET formation capacity shown as the ratio of NETs/neutrophil in LCCA cross-sections subjected to immunofluorescence staining (NaCl group = 3; PMX53 group = 4; PMX53 + AG490 group = 3; AG490 group = 4). Data are presented as mean ± SD

As stated earlier, the use of the selective C5aR1 receptor inhibitor PMX53 reduced the arterial thrombotic burden. To investigate whether C5a induces the generation of NETs by inhibiting Mito-STAT3 to promote the development of arterial thrombosis, AG490 was used to explore the effect on the reduction in arterial thrombotic burden induced by PMX53. Through pharmacologic blockade of the Mito-STAT3 pathway, AG490 abolished the reduction in arterial thrombotic burden induced by PMX53 in vivo (Fig. 5A, B, C). The reduction in thrombus weight induced by PMX53 was suppressed by AG490 (Fig. 5D). In addition, AG490 reversed the increased in blood flow induced by PMX53 in the FeCl_3_-induced thrombus mice (Fig. 5E, F). NET formation in thrombi was significantly reduced by PMX53, and AG490 reversed this reduction (Fig. 5G, H). Considering all the data discussed above, C5a induced the generation of NETs by inhibiting Mito-STAT3 to promote the development of arterial thrombosis.

### Colivelin abolished C5a-induced NETosisin vitro

**Fig. 6:**
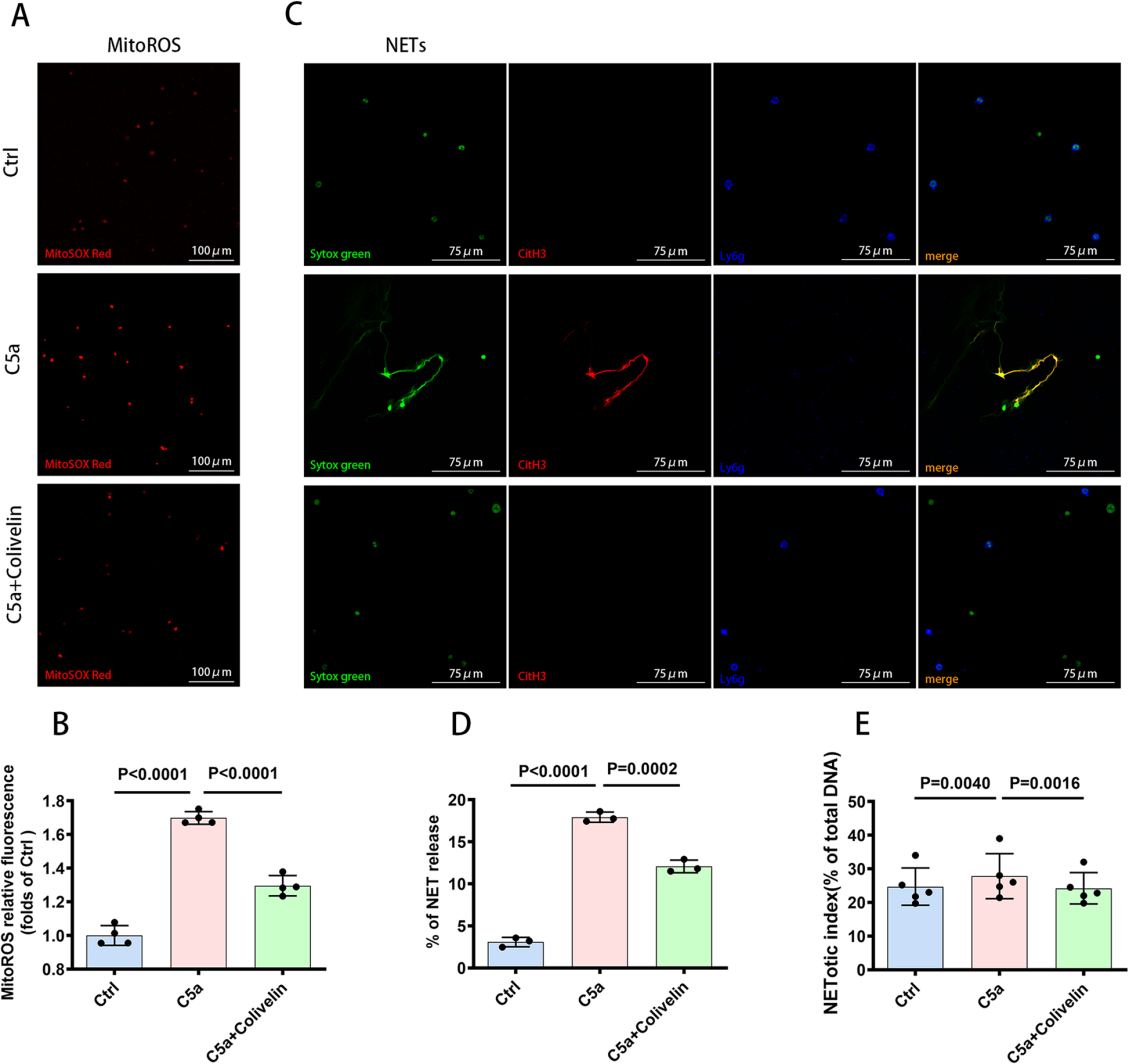
Colivelin abolished C5a-induced NETosis in vitro. **A** Fluorescence images showing ROS production in mitochondria indicated by MitoSox Red dye. Colivelin abolished the elevation of Mito-ROS levels induced by C5a in vitro. Scale bar = 100 µm. **B** Quantification of the production of Mito-ROS showed as MitoROS relative fluorescence (folds of the control group) (*n* = 4 each). **C** Representative images of immunofluorescence staining for DNA (SYTOX green), CitH3 (red), and Ly6g(blue) in vitro after stimulation with C5a showing NET formation. NET formation was attenuated by Colivelin. Scale bar = 75 µm. **D** Quantification of NET formation capacity shown as percentage of NET release in vitro, as assessed by immunofluorescence staining (*n* = 3 each). **E** NETosis was measured using a plate reader assay (*n* = 5 each). Data are presented as mean ± SD

To determine whether the activation of the Mito-STAT3 pathway is related to C5a-induced NETosis, the effect of activating the Mito-STAT3 pathway on C5a-induced NETotic capacity was investigated. As expected, in the presence of the STAT3 agonist Colivelin, the effect of C5a on the increased production of Mito-ROS was suppressed (Fig. 6A, B). In addition, the application of Colivelin reversed NETosis induction by C5a (Fig. 6C, D, E), indicating that members of the Mito-STAT3 signaling pathway are key mediators of the process of C5a-induced NETosis.

## Discussion

Thrombosis is the most frightening complication of cardiovascular diseases and a main cause of death worldwide, which makes it a major health-care challenge [4]. Antithrombotic approaches, which are widely used but confer an inherent risk of bleeding, effectively target platelets and the coagulation cascade. Nevertheless, antithrombotics cannot completely prevent thrombotic events [4]. A tight interaction between thrombosis and inflammation has been recognized in recent decades. Under certain circumstances, thrombosis is a physiological process that constitutes an intrinsic effector mechanism of innate immunity, which is termed “immunothrombosis” [3]. The mechanistic details of immunothrombosis processes are being elucidated, including inflammatory cells and cytokines participating in the coagulation cascade. The therapeutic gap may be due to inflammation, a not yet adequately addressed mechanism. As some of the molecular mediators of immunothrombosis are distinct and at least partly dispensable for normal hemostasis, targeting immunothrombosis rather than hemostasis could provide new options to treat and prevent pathological thrombosis [3]. In this study, we demonstrated that complement C5a induced the generation of NETs to promote the development of arterial thrombosis in a FeCl_3_-induced arterial thrombosis model in C57BL/6 mice. Hence, our study identifies complement C5a as a potential new target for the treatment and prevention of thrombosis.

Neutrophils are the most abundant leukocytes that form the core of our innate immunity and the first line of defense in controlling infection via oxygen-dependent and oxygen-independent mechanisms [31, 32]. The specific contribution of neutrophils to thrombus formation has only recently been highlighted. Neutrophils and NETs have been found in coronary thrombosis induced by endothelial activation[33], plaque erosion [34, 35], and rupture [36, 37]. Neutrophils and NETs also participate in thrombi derived from patients with STEMI [38–40] and thrombosed stents [41]. Neutrophils regulate thrombosis via several mechanisms in which NETs play a central role [42].

The complement system is part of the innate immune response and forms a cascade of over forty proteins, which are synthesized and secreted by a number of cells under various stimuli, including cytokines and hormones, present in plasma or bound to the target surface [43]. When C5 is cleaved, anaphylatoxins C5a, which stimulates the recruitment of inflammatory cells and induces the oxidative burst of neutrophils and macrophages, is released in the fluid phase. In classic neutrophil chemotaxis assays, C5a is potent chemotactic agent for neutrophils [44]. In acute myocardial infarction, C5a accumulates at the site of coronary thrombus and mediates neutrophil migration toward culprit site-derived plasma [17]. In vitro, C5a has also been demonstrated to be a NETosis stimulus itself alone [19] or when interferon gamma is used as a priming cytokine [45]. Considering all these findings, we hypothesize that C5a chemotactically mediates neutrophil migration toward the culprit site and the formation of NETs to participate in artery thrombosis. Our data confirmed that the plasma concentrations of C5a was higher in patients with STEMI than in patients with angina. In the FeCl_3_-induced thrombus model, we detected elevated expression of C5a in the plasma. Meanwhile, with inhibition of C5aR1 receptor by PMX53, the thrombus area and weight were decreased, and the declination of both blood flow in the vessel lumen and NET formation in thrombi were attenuated. Blockade of the interaction between C5a and neutrophils reduced NET formation and the thrombus burden, indicating that C5a may be the primary trigger for the ejection of NETs in artery thrombosis.

There is a consensus that microbial agents, biochemical stimuli, calcium influx, immune complexes, or contact with platelets can trigger NET formation [46–52]. The composition of NETs varies depending on the stimulus, and different pathways may generate NETs with different functional attributes [53]. Different agonists induce different forms of ROS (NADPH oxidase-ROS or Mito-ROS), which are thought to be critical for NET formation [25]. The process by which C5a promotes neutrophil ejection of NETs has not yet been explored. In this study, we showed that NET formation induced by C5a was dependent on Mito-ROS.

To explore how C5a mediates Mito-ROS production, we focused on the pathway regulating Mito-ROS. STAT3 was originally found to be a transcription factor bound to genes. After the discovery that STAT3 is also present in mitochondria (mitochondrial STAT3), STAT3 has also been proposed to reduce Mito-ROS production by increasing membrane polarization and ATP production, inducing the formation of respiratory supercomplexes in the mitochondria, minimizing electron leakage during transport in the ETC and enhancing the activity of lactate dehydrogenase [27, 54–59]. C5a stimulated the phosphorylation of STAT3 at Ser^727^ but not at Tyr^705^in the NR8383 macrophage cell line, for which cell lysates containing total STAT3 (nuclear STAT3 and Mito-STAT3) were analysed [60]. The effect of C5a on the phosphorylation of Mito-STAT3 at Ser^727^ was explored in this study. Unexpectedly, C5a reduced the phosphorylation of Mito-STAT3 at Ser^727^ in neutrophil-like cells, through which C5a induced the production of Mito-ROS. The inhibition of Mito-STAT3 by AG490 abolished the reduction in NET and thrombus formation induced by PMX53, indicating that C5a triggered NETosis to participate in thrombosis via Mito-STAT3.

## Conclusions

**Fig. 7:**
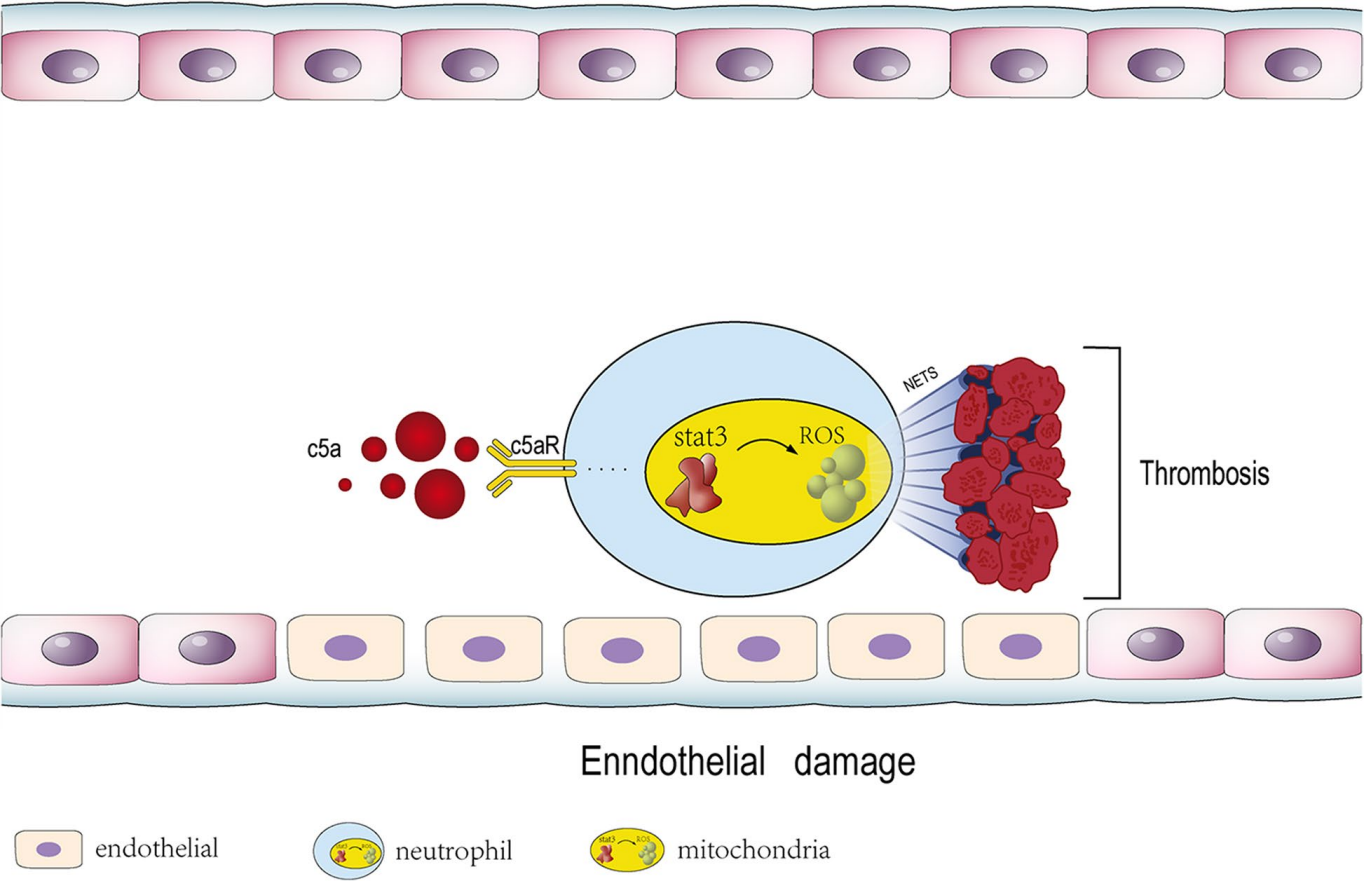
Visual summary: the effect of complement C5a in arterial thrombosis. In arterial thrombosis, C5a chemotactically attracts neutrophils to migrate towards the culprit site and triggers the release of NETs, which contribute to thrombosis by promoting coagulation and stabilizing clots. C5a-induced promotion of NET release is dependent on Mito-ROS production. C5a induces the Mito-ROS production by inhibiting mitochondrial STAT3 activity

In summary, our data suggest that via inhibiting Mito-STAT3 to elicit Mito-ROS generation, C5a triggers the generation of NETs to promote the development of arterial thrombosis. The interplay between C5a and neutrophils could be a potential target for anticoagulation (Fig. 7).

## Supplementary Information

**Additional file 1.** **Table 1**. Characteristics of patients with STEMI. **Table 2**. Characteristics of patients with angor pectoris. **Figure S1.** Raw WB data.

## Abbreviations

NETs: Neutrophil extracellular traps
LCCA: Left common carotid artery
LICA: Left internal carotid artery
LECA: Left external carotid artery
ROS: Reactive oxygen species
Mito-ROS: Mitochondrial ROS
MI: Myocardial infarction
PAD4: Peptidylarginine deiminase 4
NADPH: Nicotinamide adenine dinucleotide phosphate
NE: Neutrophil elastase
PMA: Phorbol-12-myristate-13-acetate
DVT: Deep vein thrombosis
FeCl_3_: Ferric chloride
CitH3: Citrullinated histone H3
STEMI: ST-elevation myocardial infarction
STAT3: Signal Transducers and Activators of Transcription family 3
Mito-STAT3: Mitochondrial STAT3
DMSO: Dimethyl sulfoxide
