# Supplementary material for: Complement C5a induces the generation of neutrophil extracellular traps by inhibiting mitochondrial STAT3 to promote the development of arterial thrombosis
Source: Thromb J. 2022 Apr 29;20:24. doi: 10.1186/s12959-022-00384-0 (PMC9051782; doi:10.1186/s12959-022-00384-0)

**Thrombosis Journal**

**Complement C5a induces the generation of neutrophil extracellular traps by inhibiting mitochondrial STAT3 to promote the development of** **arterial thrombosis**

Yejia Chen,^1, #^ Xiaobo Li,^1, #^ Xinxin Lin,^1^ Hongbin Liang,^1^ Xuewei Liu,^1^

Xinlu Zhang,^1^ Qiuxia Zhang,^1^ Fengyun Zhou,^1^ Chen Yu,^1^ Li Lei,^1^

and Jiancheng Xiu^1, *^

^1, *^Department of Cardiology, State Key Laboratory of Organ Failure Research, Nanfang Hospital, Southern Medical University, Guangzhou 510515, Guangdong, China

^*^Correspondence address: Jiancheng Xiu, MD, PhD, Department of Cardiology, Nanfang Hospital, Southern Medical University, Guangzhou 510515, Guangdong, China. Tel : +86-13903064940; Fax: +86-20-61641049; E-mail: [xiujch@163.com](mailto:xiujch@163.com)

**SUPPLEMENTAL MATERIAL**

**TABLE 1**. Characteristics of patients with STEMI

| Patient | Sex | Age(Y) | height(M) | Weight(Kg) | Neutrophil(10^9/L) | Emergency PCI | Coronary thrombus shadow | C5a(ng/ml) |
| --- | --- | --- | --- | --- | --- | --- | --- | --- |
|  |  |  |  |  |  |  |  |  |
| STEMI 1 | F | 64 | 158 | 66 | 4.88 | YES | NO | 12.09 |
| STEMI 2 | M | 76 | 169 | 80 | 8.10 | YES | YES | 10.78 |
| STEMI 3 | F | 83 | 158 | 55 | 15.54 | YES | YES | 10.65 |
| STEMI 4 | F | 55 | 156 | 50 | 11.19 | YES | YES | 11.27 |
| STEMI 5 | M | 61 | 166 | 70 | 7.40 | YES | YES | 10.45 |
| STEMI 6 | M | 49 | 168 | 72 | 8.35 | YES | YES | 12.02 |
| STEMI 7 | M | 59 | 170 | 80 | 6.09 | YES | NO | 14.47 |
| STEMI 8 | M | 49 | 165 | 74 | 16.31 | YES | YES | 11.45 |
| STEMI 9 | M | 53 | 164 | 68 | 4.38 | YES | NO | 12.07 |
| STEMI 10 | M | 68 | 172 | 75 | 3.95 | YES | NO | 12.58 |
| STEMI 11 | F | 49 | 156 | 50 | 7.70 | YES | YES | 10.85 |
| STEMI 12 | M | 45 | 169 | 68 | 3.26 | YES | NO | 11.79 |
| STEMI 13 | F | 68 | 155 | 62 | 8.38 | YES | NO | 10.75 |
| STEMI 14 | M | 44 | 175 | 88 | 4.96 | YES | YES | 14.30 |
| STEMI 15 | M | 65 | 169 | 64 | 7.96 | YES | YES | 12.95 |
|  |  |  |  |  |  |  |  |  |

*STEMI, ST-elevation myocardial infarction; F, Female; M, male; PCI, Percutaneous Transluminal Coronary Intervention.*

**TABLE 2**. Characteristics of patients with angor pectoris

| Patient | Sex | Age(Y) | height(M) | Weight(Kg) | Neutrophil(10^9/L) | PCI | Coronary thrombus shadow | C5a(ng/ml) |
| --- | --- | --- | --- | --- | --- | --- | --- | --- |
|  |  |  |  |  |  |  |  |  |
| AP1 | M | 54 | 172 | 68 | 2.94 | YES | NO | 7.13 |
| AP2 | M | 55 | 169 | 77 | 3.12 | YES | NO | 7.93 |
| AP3 | F | 57 | 152 | 58 | 3.12 | YES | NO | 9.09 |
| AP4 | M | 58 | 169 | 70 | 5.47 | YES | NO | 11.96 |
| AP5 | M | 48 | 166 | 66 | 4.12 | YES | NO | 8.47 |
| AP6 | F | 68 | 160 | 60 | 4.62 | YES | NO | 11.81 |
| AP7 | F | 59 | 155 | 68 | 1.54 | YES | NO | 7.60 |
| AP8 | M | 61 | 176 | 72 | 3.17 | YES | NO | 10.75 |
| AP9 | F | 54 | 154 | 58 | 4.74 | YES | NO | 10.59 |
| AP10 | M | 48 | 170 | 80 | 5.83 | YES | NO | 10.22 |
| AP11 | M | 67 | 172 | 72 | 2.70 | YES | NO | 11.09 |
| AP12 | M | 67 | 174 | 66 | 2.37 | YES | NO | 7.12 |
| AP13 | M | 62 | 166 | 62 | 3.21 | YES | NO | 10.61 |
| AP14 | M | 76 | 162 | 68 | 2.91 | YES | NO | 9.31 |
| AP15 | M | 64 | 168 | 78 | 3.21 | YES | NO | 10.65 |
|  |  |  |  |  |  |  |  |  |

*AP,angor pectoris; F, Female; M, male; PCI, Percutaneous Transluminal Coronary Intervention.*

**Figure S1 Raw WB data**


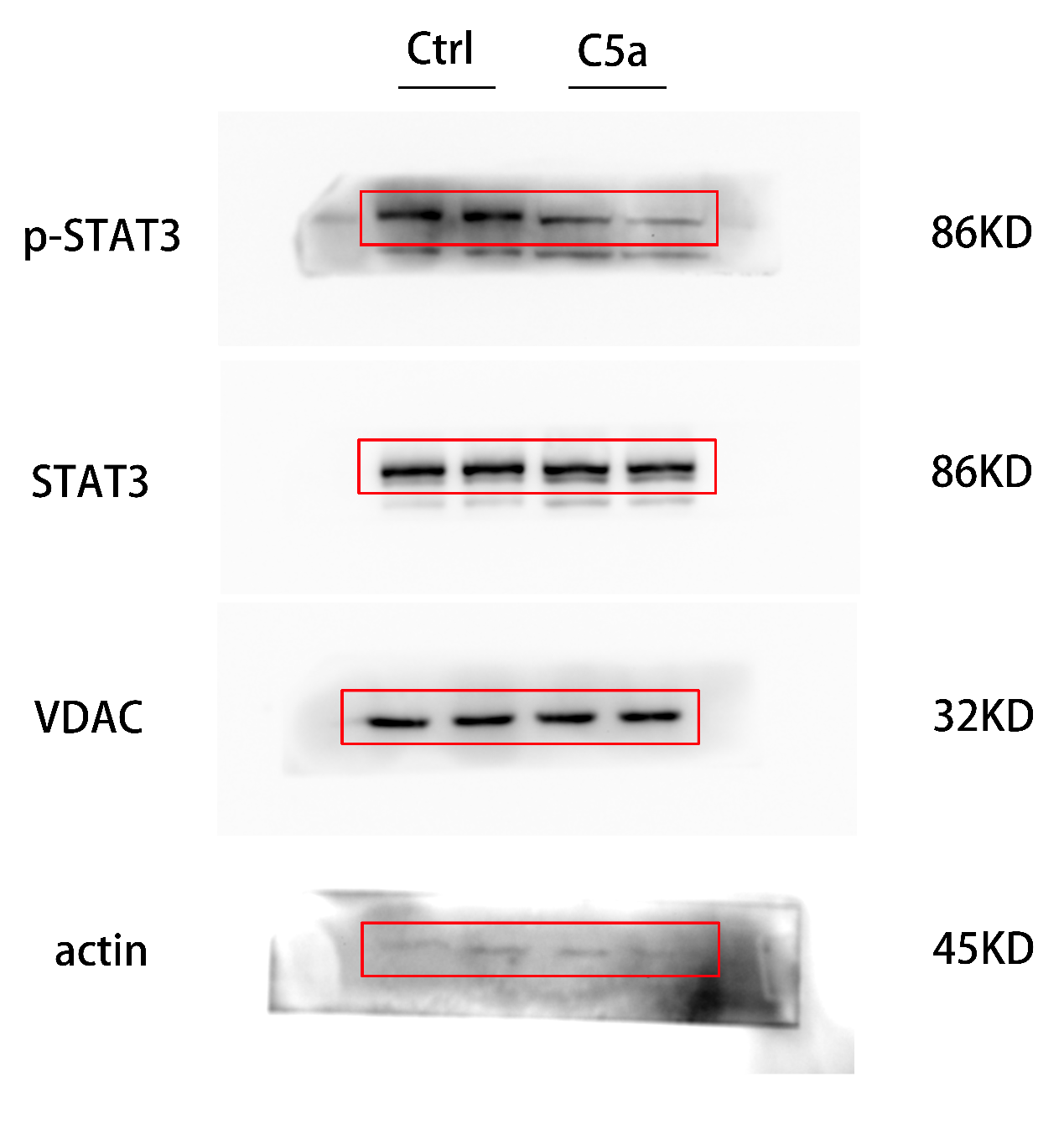

Supplement: Supplementary file 1 — Additional file 1. Table 1. Characteristics of patients with STEMI. Table 2. Characteristics of patients with angor pectoris. Figure S1. Raw WB data. [file 12959_2022_384_MOESM1_ESM.docx]
